# Supplementary material for: Cholesterol‐loaded nanoparticles ameliorate synaptic and cognitive function in Huntington's disease mice
Source: EMBO Mol Med. 2015 Nov 20;7(12):1547–64. doi: 10.15252/emmm.201505413 (PMC4693506; doi:10.15252/emmm.201505413)
Supplement: Supplementary file 1 — Appendix [file EMMM-7-1547-s001.pdf]

Appendix PDF

Cholesterol-loaded nanoparticles ameliorate synaptic and cognitive function in Huntington's disease mice

Marta Valenza[1§], Jane Y. Chen[2§], Eleonora Di Paolo[1¶], Barbara Ruozi[3¶], Daniela Belletti[3], Costanza Ferrari Bardile[1], Valerio Leoni[4,5], Claudio Caccia[4], Elisa Brilli[1], Stefano Di Donato[4], Marina M. Boido[6], Alessandro Vercelli[6], Maria A. Vandelli[3], Flavio Forni[3], Carlos Cepeda[2], Michael S. Levine[2] , Giovanni Tosi[3], Elena Cattaneo[1]

[1]Department of BioSciences and Centre for Stem Cell Research, Università degli Studi di Milano, Milan, Italy; [2]Intellectual and Developmental Disabilities Research Center, Semel Institute for Neuroscience, Brain Research Institute, David Geffen School of Medicine, University of California Los Angeles, Los Angeles, CA; [3]Department of Life Sciences, University of Modena and Reggio Emilia, Modena, Italy. [4]Neurological Institute C. Besta, Milan, Italy. [5]Laboratory of Clinical Chemistry, Ospedale di Circolo e Fondazione Macchi, Varese, Italy. [6]Neuroscience Institute Cavalieri Ottolenghi Neuroscience Institute of Turin, Orbassano (Turin), Italy.
§co-first authors. ¶co-second authors

Content:

Appendix Figures S1-S11
Appendix Tables S1-S7
Appendix Methods

Appendix Figure S1

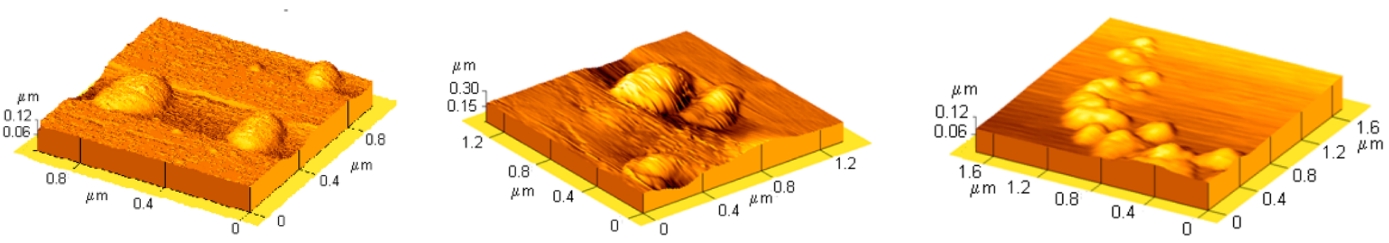

Rhod-NPs　　Rhod-g7-NPs　　Rhod-g7-NPs-NBD-Chol1

Appendix Figure S1. AFM analysis (3D elaboration) of Rhodamine Samples (Rhod-NPs, Rhod-g7-NPs, Rhod-g7-NPs-NBD-Chol1). The chemico-physical properties have been described in Appendix Table 2.

Appendix Figure S2

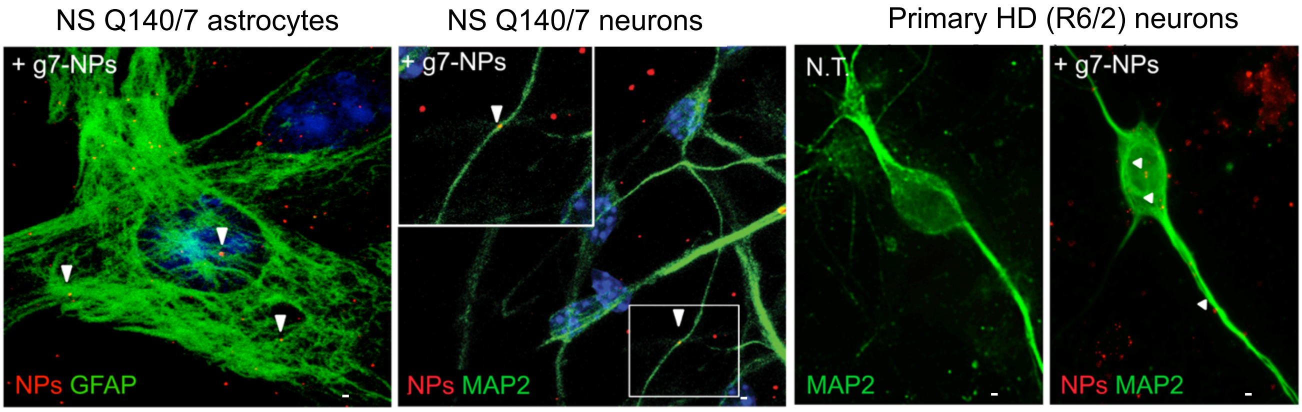

Appendix Figure S2. g7-NPs are taken up *in vitro* by different brain cells expressing mutant huntingtin. (a) high-magnification confocal image (cropped) of astrocytes at day 14 of glial differentiation derived from NS Q140/7 cells incubated with g7-NPs for 6 hrs and immunostained for GFAP. Original magnification: 63x. (b) Representative high-magnification confocal image (cropped) of neurons at day 7 of neuronal differentiation derived from NS Q140/7 cells incubated with g7-NPs for 6 hrs and immunostained for MAP2. The boxed region is shown at higher magnification in the inset. Original magnification: 63x. (c) Representative high-magnification confocal image (cropped) of primary cortical neurons generated from R6/2 embryos at 18 days of gestation, not treated (N.T.) or treated with g7-NPs (+g7-NPs) for 6 hrs and immunostained for MAP2. Original magnification: 63x. Hoechst 33258 was used to counterstain the nuclei. White arrowheads indicate intracellular g7-NPs. Scale bar: 5 μm.

Appendix Figure S3

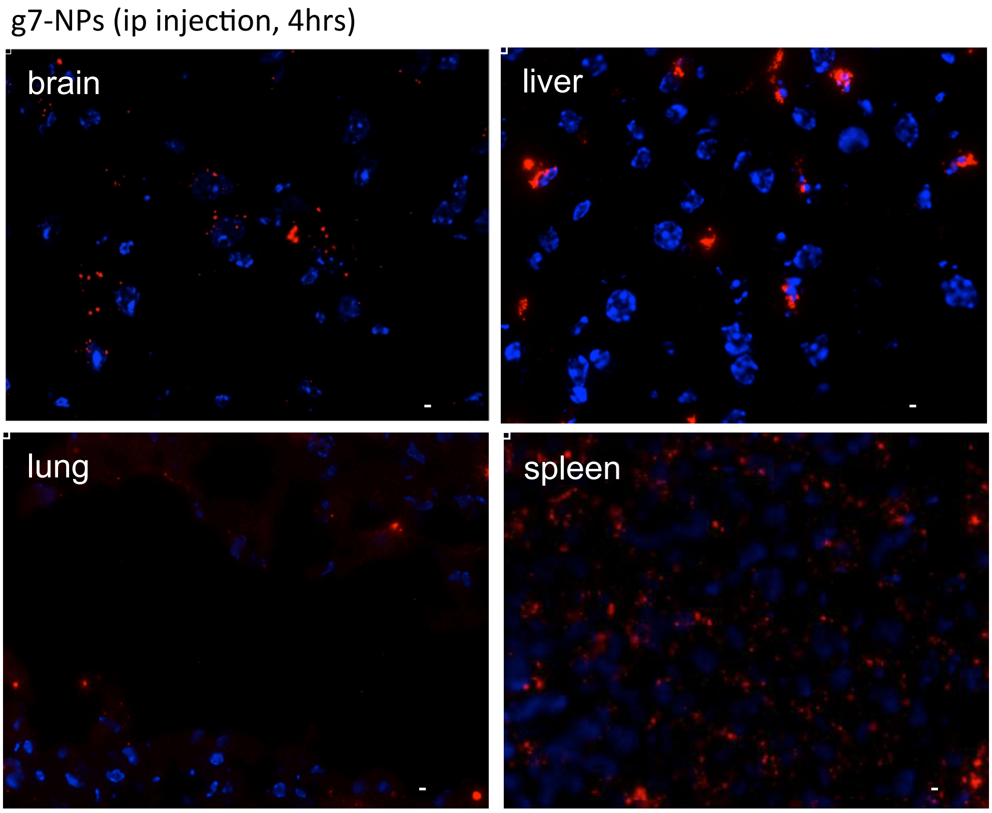

Appendix Figure S3. Distribution of g7-NPs in brain and peripheral tissues in mice. Representative high-magnification confocal images of brain, liver, lung, and spleen slices from WT mice ip injected with g7-NPs and sacrificed after 4 hrs. 4',6-diamidino-2-phenylindole was used to counterstain nuclei. Original magnification: 63x (scale bar: 10μm).

Appendix Figure S4

Hippocampus, 24h post-injection – low magnification

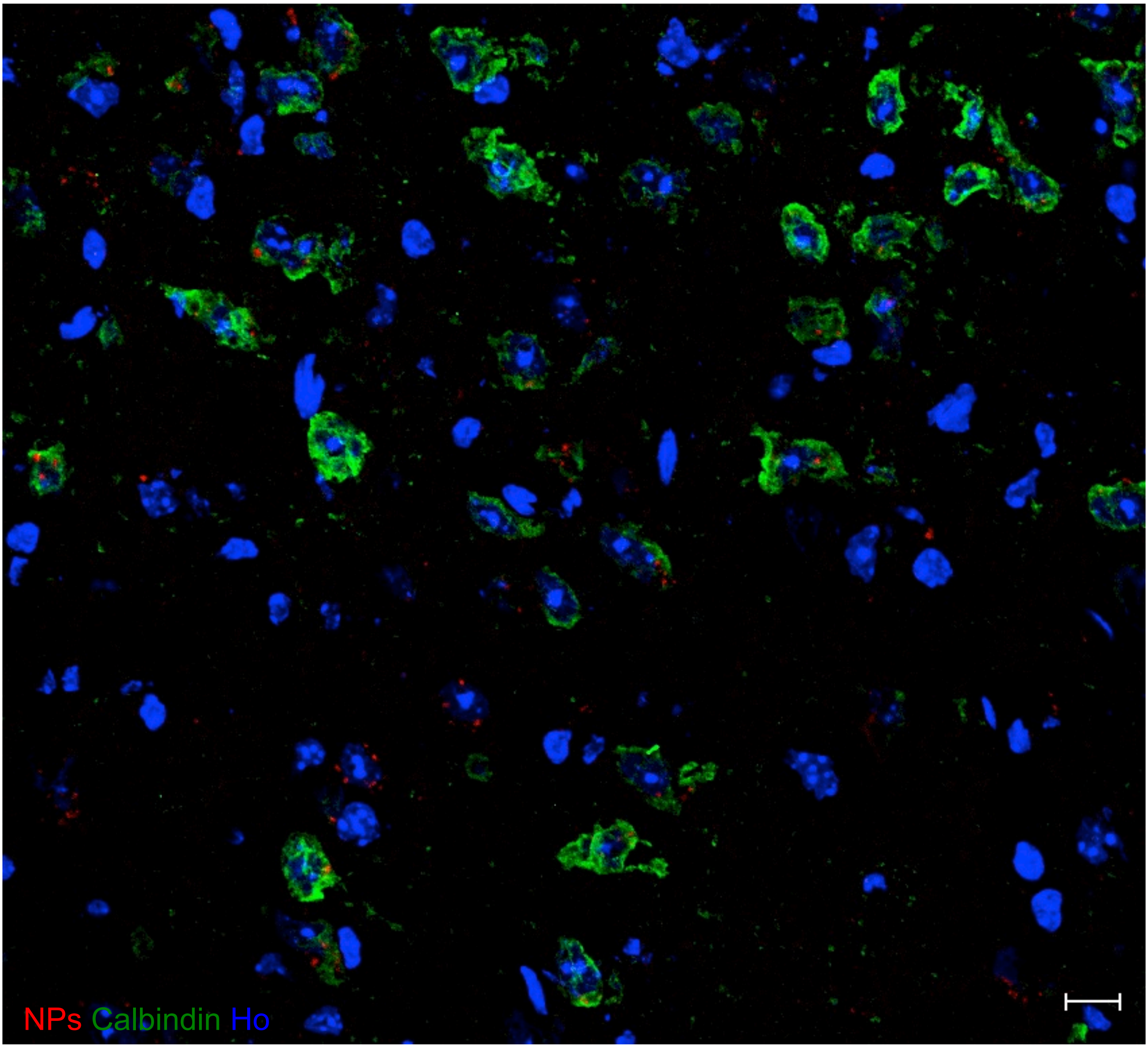

Appendix Figure S4. Distribution of g7-NPs in calbindin-positive neurons in hyppocampus. Representative confocal image of brain slice from R6/2 mice ip injected with g7-NPs and sacrificed after 24 hrs. 4',6-diamidino-2-phenylindole was used to counterstain nuclei. Scale bar: 5 µm.

Appendix Figure S5

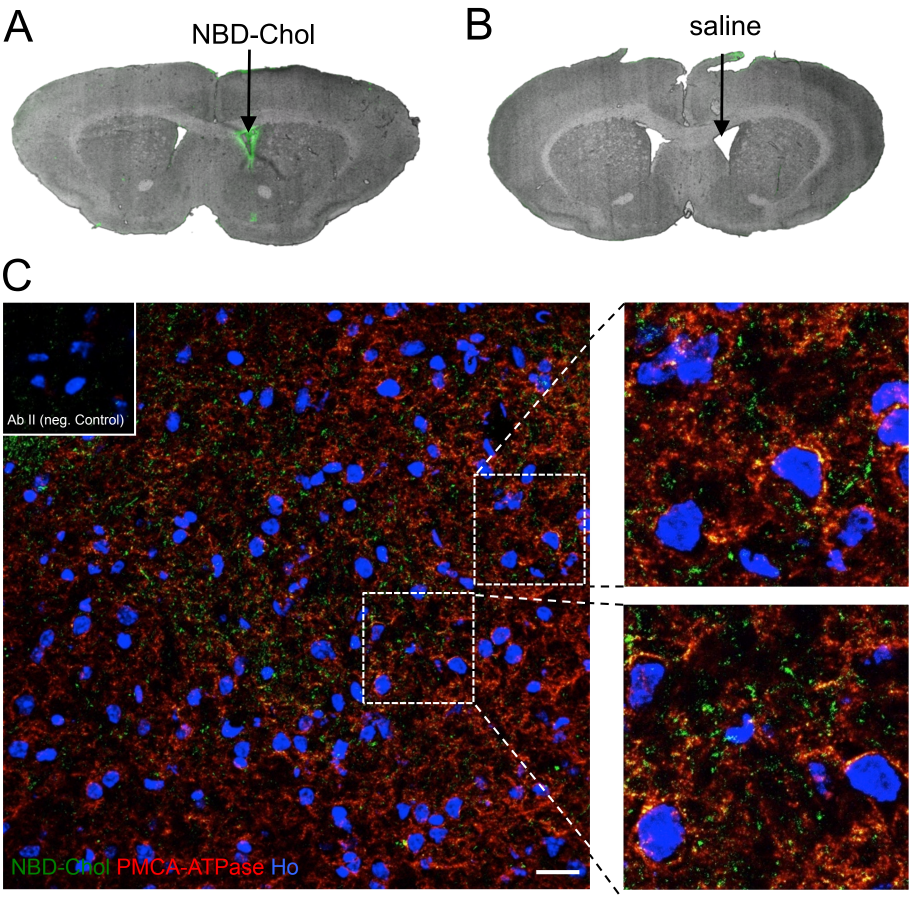

Appendix Figure S5. Co-localization of NBD-Chol with PMCA ATPase *in vivo*. Representative confocal images of brain slice from WT mice injected with NBD-Chol (10ug) into the brain ventricles and sacrificed after 7 hrs. A-B) low-magnification images of brain slices of mice injected with NBD-Chol (A) or with saline (B) into the ventricle (see arrows). C) Representative 63x confocal image of brain slices of WT mice injected with NBD-Chol (green) and immunostained against PMCA ATPase (red), a marker for plasma membrane (scale bar: 10µm). In the crops, it is possible to appreciate the distribution of NBD-Chol into brain cells' membrane (yellow signal). The **Hoechst** 33342 dye (Ho; blue) was used to counterstain nuclei.

Appendix Figure S6

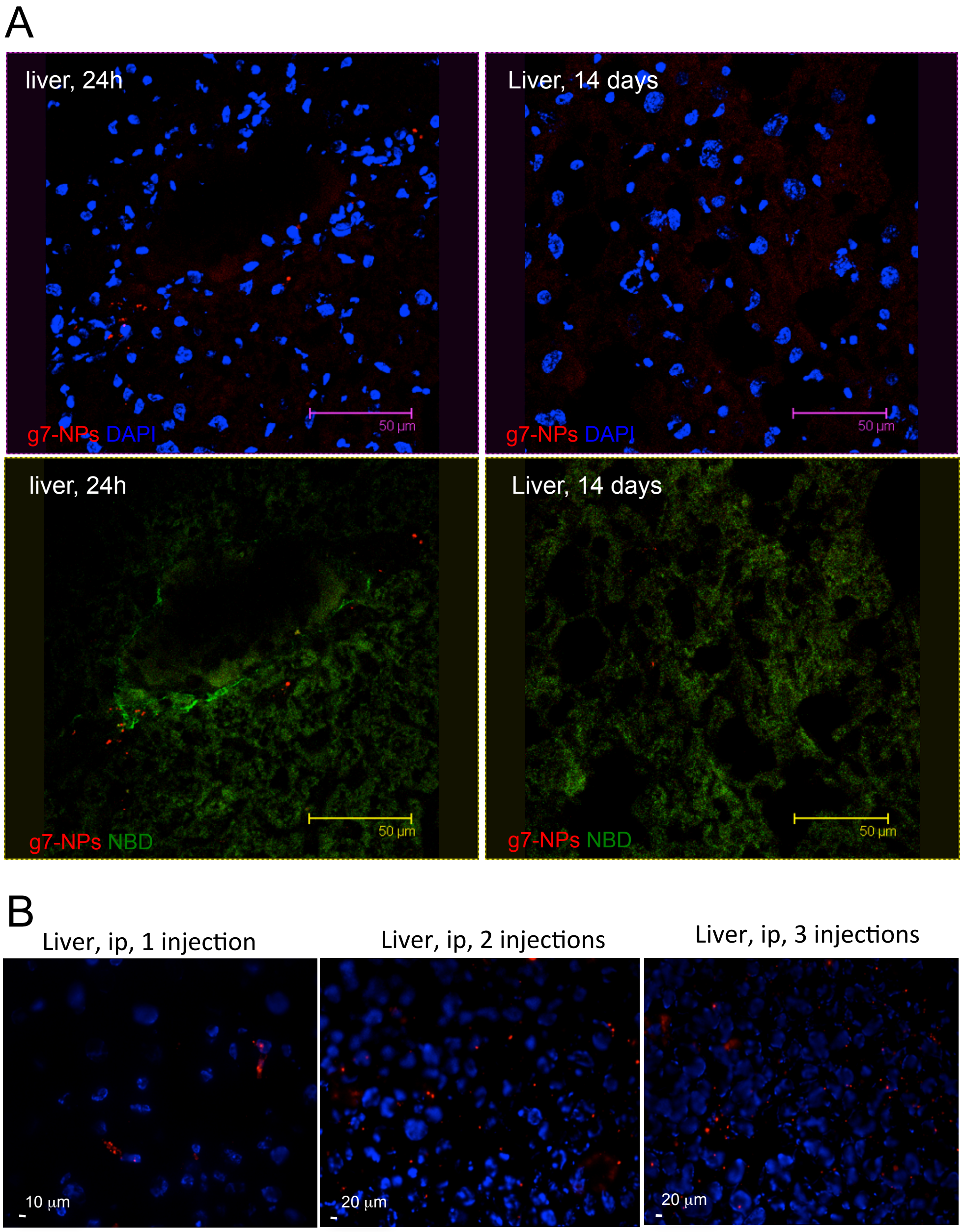

Appendix Figure S6. Kinetics of g7-NPs in the brain and liver. (a-b) Representative confocal images of brain slices (a) and of liver slices (b) from R6/2 mice ip injected with NBD-chol-g7-NPs and sacrificed after 7 days (a) or after 24 hrs or 14 days (in b; the same experiment described in Fig. 1i-l). (c) Confocal images of liver slices from WT mice after a single or multiple injections of g7-NPs and sacrificed after 1 week. Scale bars: 10 µm (in a), 50 µm (in b), and 10-20 µm (in c). All images suggest a faster degradation of g7-NPs in the liver than in the brain. 4',6-diamidino-2-phenylindole (DAPI) was used to counterstain nuclei.

Appendix Figure S7

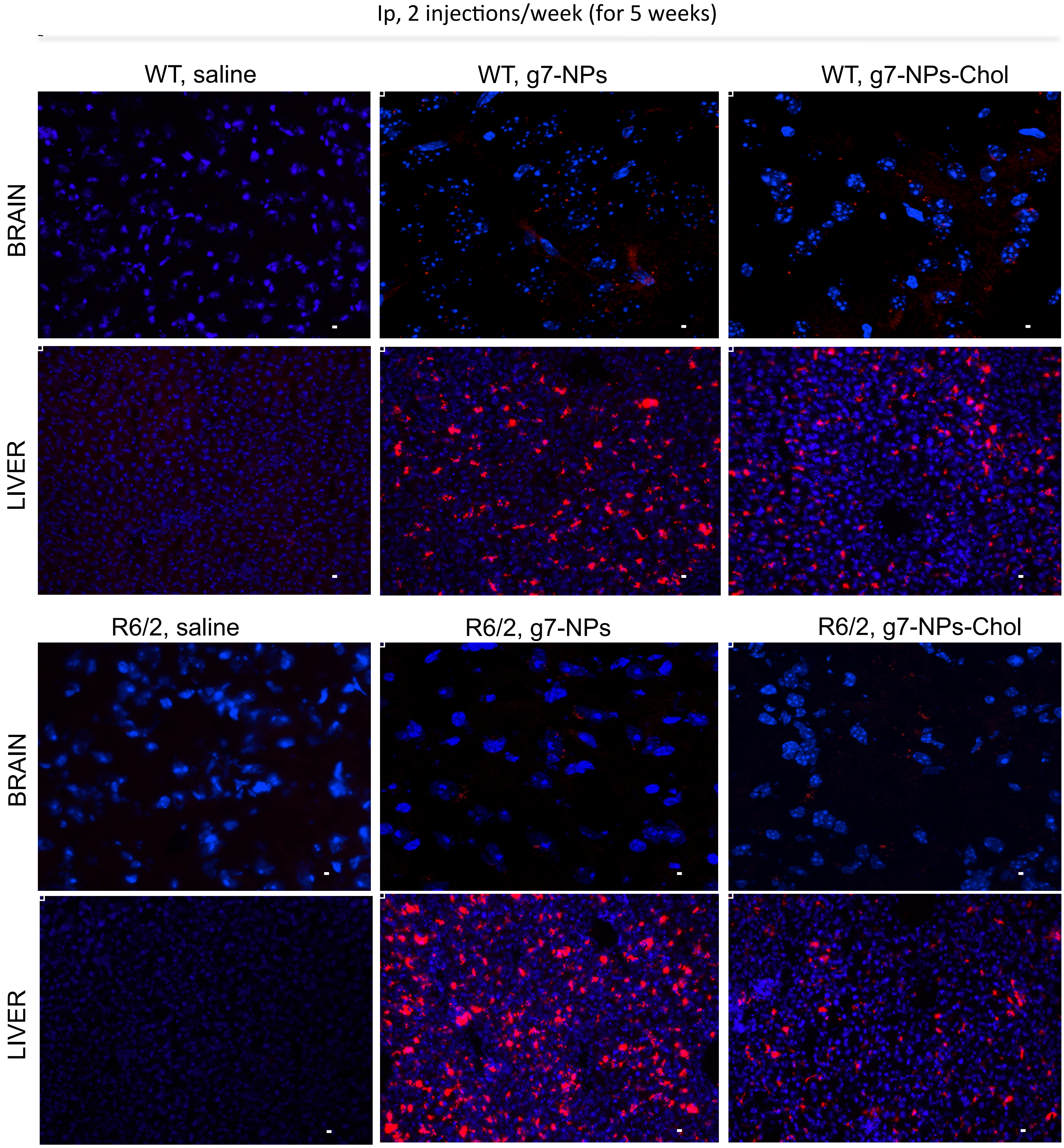

Appendix Figure S7. Distribution of g7-NPs-Chol or empty g7-NPs in the brain and liver of WT and HD mice in which electrophysiological parameters were analyzed. The presence of g7-NPs was analyzed by fluorescent microscopy in the liver and in cortical pieces taken from brains before electrophysiological analysis. 4',6-diamidino-2-phenylindole was used to counterstain nuclei. Scale bars: 10μm (brain) and 50μm (liver).

Appendix Figure S8

A

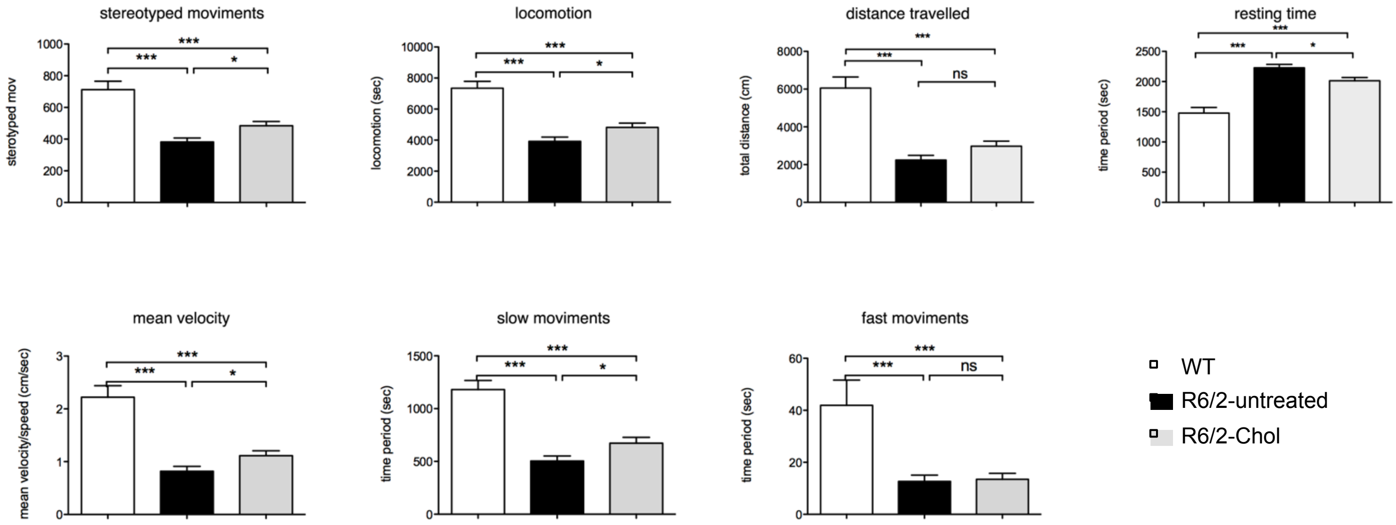

B

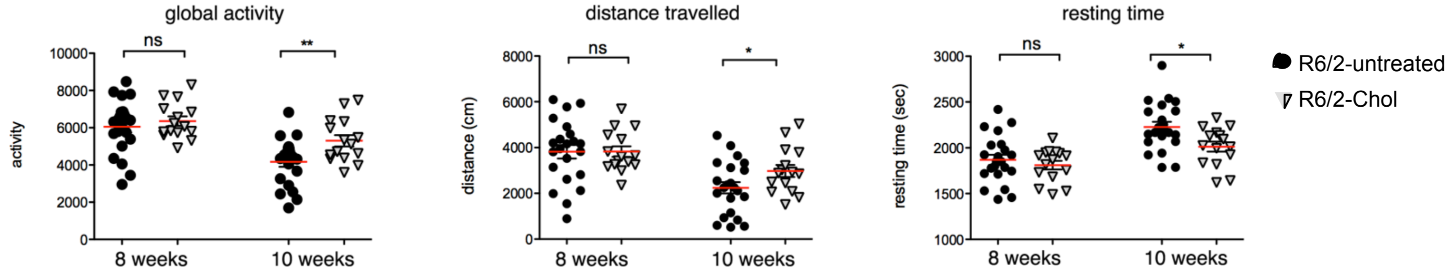

Appendix Figure S8. A) Open field parameters in WT (n=14), R6/2-untreated (n=22) and R6/2-Chol (n=15) mice. Stereotyped moviments, locomotion, total distance travelled, resting time, mean velocity, slow moviments and fast moviments at 10 weeks of age. **B)** Global activity, distance, resting time in R6/2-untreated (n=22) and R6/2-Chol mice (n=15). Data were combined to highlight the differences between R6/2 groups at each time points. All data are presented as mean ± s.e.m.; P<0.05 was determined by one-way ANOVA followed by Newmann-Keuls multiple comparison test or t-student test .

Appendix Figure S9

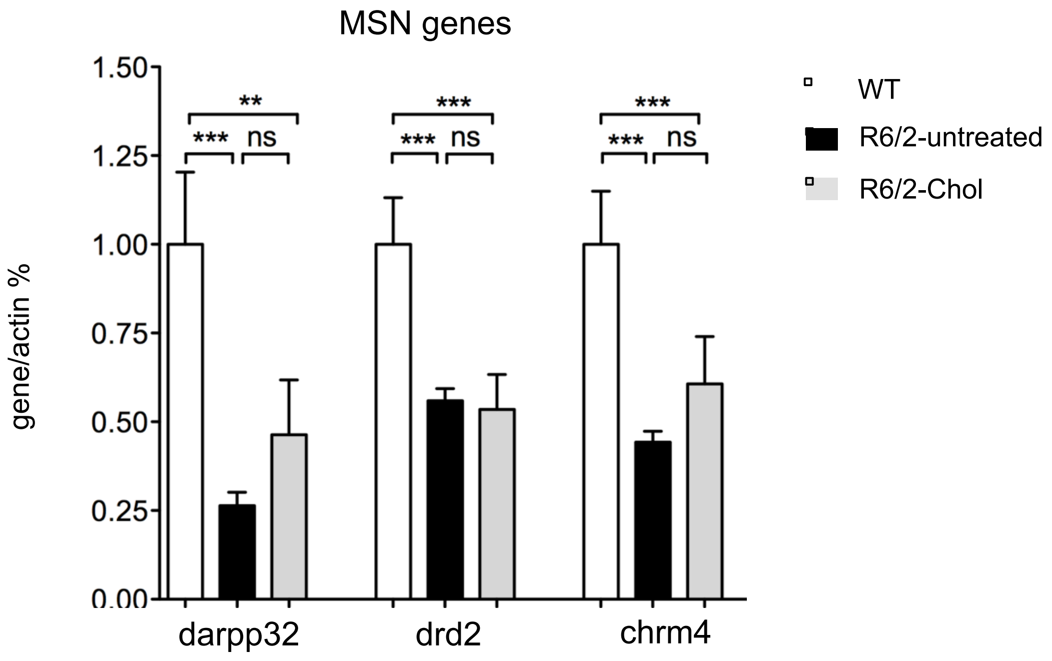

Appendix Figure S9. mRNA levels of striatal genes considered hallmarks of Medium Spiny Neurons (MSNs), such as darpp32, drd2 and chrm4. Data are presented as mean ± s.e.m.; WT (n=4), R6/2-untreated (n=7) and R6/2-Chol animals (n=3) at 12 weeks of age. P<0.05 was determined by one-way ANOVA followed by Newmann-Keuls multiple comparison tests.

Appendix Figure S10

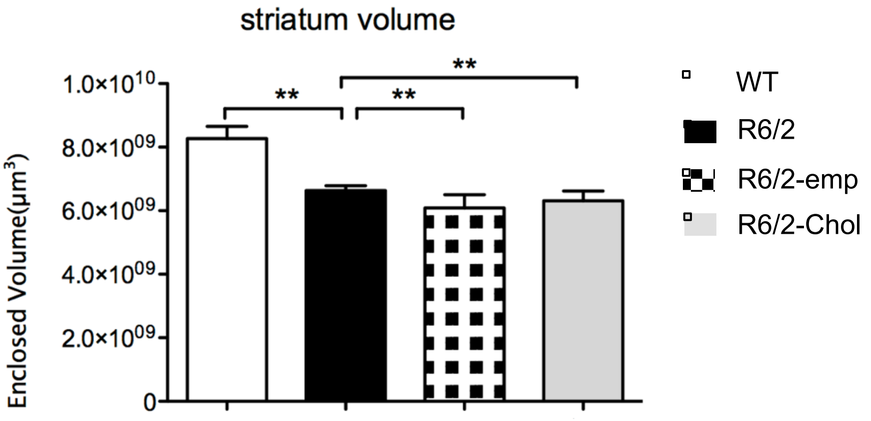

Appendix Figure S10. Striatal volume was evaluated by Neurolucida Analysis at 12 weeks of age in WT (n= 7), R6/2 (n=7), R6/2-emp (n=6), R6/2-Chol (n=8). Data represent mean ± SEM. $P<0.05$ was determined by one-way ANOVA followed by Newmann-Keuls multiple comparison tests.

Appendix Figure S11

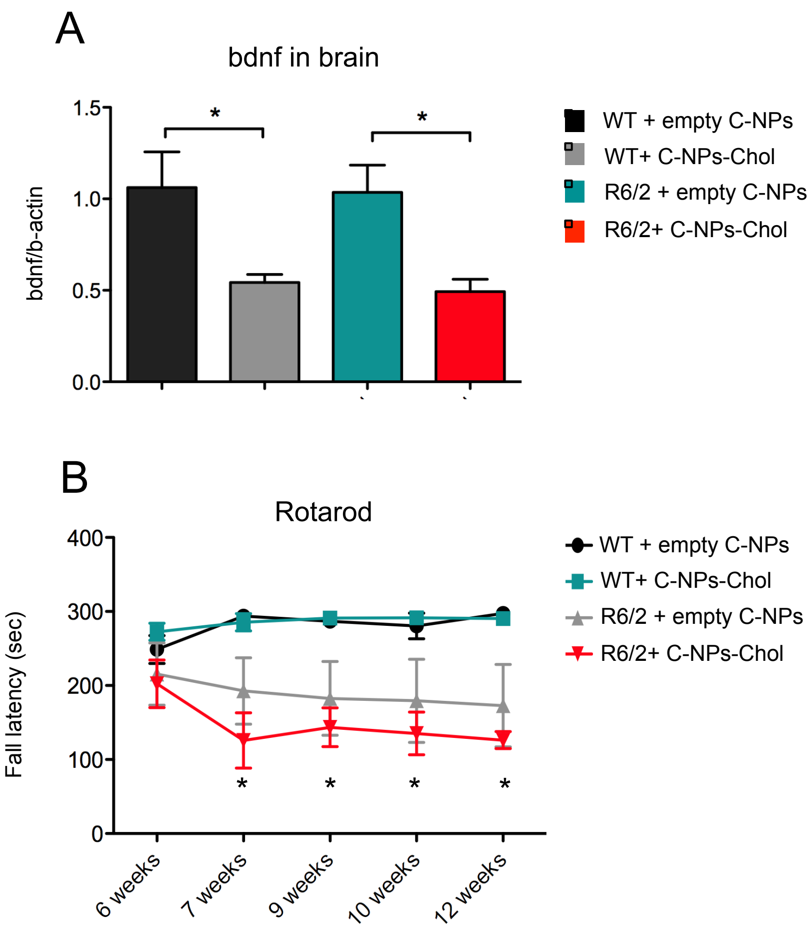

Appendix Figure S11. Initial trial performed in WT and R6/2 mice treated with unmodified control NPs (not able to cross the BBB) loaded with cholesterol (C-NPs-Chol) (n=4/genotype) or with empty C-NPs (n=4/genotype) according to the same experimental paradigm shown in Figure 5. **A)** mRNA levels of BDNF, a brain-specific hallmark of HD, were found reduced in the brain of R6/2 mice independently from the treatment, suggesting that peripheral cholesterol supplementation did not influence BDNF expression in the HD brain, differently from that observed in R6/2 mice treated with g7-NPs-Chol (Figure 6C). **B)** behavioral performance, as measured by rotarod test, was not improved in R6/2 treated with C-NPs-Chol compared to WTs; similarly, peripheral cholesterol supplementation via C-NPs-Chol injection did not influence rotarod performance in R6/2 mice (see R6/2+C-NPs-Chol line versus R6/2+ empty C-NPs). Data represent mean ± SEM. $P<0.05$ was determined by one-way ANOVA followed by Newmann-Keuls multiple comparison tests. On this basis, the unmodified control NPs loaded with cholesterol (C-NPs-Chol) group was no longer included in subsequent trials with g7-NPs-Chol.

Appendix Table S1. Composition of NPs involved in the study.

The amounts (mg) of polymers (PLGA503H; Rhod-PLGA502H; g7-PLGA), of cholesterol (Chol) and of labeled cholesterol (NDB-Chol) used as starting materials are reported for each formulation.

Sample	description	defined in the text as	PLGA503H (mg)	Rhod-PLGA502H (mg)	g7-PLGA503H (mg)	Chol (mg)	NBD-Chol (mg)
u-NPs	unloaded (empty) NPs	u-NPs	100				
NPs-Chol1	NPs loaded with 1mg Chol	NPs-Chol1	100			1	
NPs-Chol2	NPs loaded with 5mg Chol	NPs-Chol2	100			5	
NPs-Chol3	NPs loaded with 10mg Chol	NPs-Chol3	100			10	
NPs-NBD-Chol1	NPs loaded with 1mg NBD-Chol	NPs-NBD-Chol	100				1
Rhod-u-NPs	unloaded (empty) NPs labelled with Rhodamine (used in vivo experiments)	C-NPs	80	20			
Rhod-g7-NPs	unloaded (empty) NPs modified with g7 and labelled with Rhodamine (used in vivo experiments)	g7-NPs	70	10	20		
Rhod-g7-NPs-Chol1	NPs modified with g7, labelled with Rhodamine and loaded with NBD-chol (used in vivo experiments)	g7-NPs-NBD-Chol	70	10	20	1	
Rhod-g7-NPs-NBDChol1	NPs modified with g7, labelled with Rhodamine and loaded with NBD-chol (used in vivo experiments)	g7-NPs-Chol	70	10	20		1

Appendix Table S2. chemical-physical properties of NPs involved in the study

Dimensions of samples is expressed with Z-Average values (nm); Homegeneity of the samples is expressed as Polydispersity Index (PDI); intensity distribution is expressed as Di (nm); surface charge is expressed as ζ-pot (mV); percentage of polyvynyl alchol residual is expressed as percentage as %PVA; drug loading is expressed as mg of Chol per 100 mg of formulation (LC%) and as percentual encapsulation efficiency (EE%). Standard Deviation is reported as SD.

Sample	Chol/PLGA (mg/mg)	Z-Average nm (± S.D.)	PDI value (± S.D.)	D(i)10 nm (± S.D.)	D(i)50 nm (± S.D.)	D(i)90 nm (± S.D.)	ζ-pot mV (± S.D.)	% PVA residual (± S.D.)	mg Chol/100 mg form. LC%	EE%
u-NPs		181±11	0.08±0.01	139±12	187±12	205±12	-12±3	4.8±1.2		
NPs-Chol1	1:100	192±15	0.09±0.01	136±11	195±12	283±23	-9±4	5.1±0.4	0.7±0.1	68±5
NPs-Chol2	5:100	187±17	0.11±0.02	118±13	177±15	269±28	-8±4	4.5±1.8	0.8±0.03	17±1
NPs-Chol3	10:100	242±52	0.28±0.03	111±21	256±76	378±78	-12±10	5.8±1.0	2.4±0.3	24±3
NPs-NBD-Chol1	1:100	178±19	0.07±0.02	125±3	186±10	280±7	-6±3	5.7±0.2	0.4±0.1	44±1

Appendix Table S3 - passive membrane properties of MSNs from WT, R6/2-untreated and R6/2-chol mice

Capacitance (pF), Input Resistance (MΩ) and Time constant (ms) registered in MSNs from WT mice treated with saline (WT), R6/2 mice treated with saline or with empty g7-NPs (R6/2-untreated) and R6/2 treated with g7-NPs-Chol (R6/2-Chol).

group	Capacitance (pF)	Input Resistance (MΩ)	Time constant (ms)
WT	68.7 ± 2.27	51.6 ± 2.73	1.9 ± 0.07
R6/2-untreated	59.8 ± 2.40*	179.3 ± 14.91***	1.9 ± 0.09
R6/2-Chol	62.9 ± 3.60	180.2 ± 24.27***	1.4 ± 0.12***###

***R6/2-Chol vs WT; ### R6/2-chol vs R6/2-untreated

Appendix Table S4 - kinetics of IPSCs (A) and of EPSCs (B) in MSNs from WT, R6/2-untreated and R6/2-chol mice

A. IPSC event kinetics

group	Rise time (ms)	Decay time (ms)	Half-amplitude duration (ms)
WT-sal	3.1 ± 0.09	19.0 ± 0.62	18.3 ± 0.42
R6/2-untreated	2.7 ± 0.12	16.1 ± 0.82*	15.8 ± 0.5***
R6/2-Chol	2.5 ± 0.14***	14.2 ± 1.09***	14.7 ± 0.73***

B. EPSC event kinetics

group	Rise time (ms)	Decay time (ms)	Half-amplitude duration (ms)
WT-sal	1.0 ± 0.03	3.5 ± 0.08	4.9 ± 0.12
R6/2-untreated	1.1 ± 0.06	3.5 ± 0.15	4.7 ± 0.17
R6/2-Chol	1.0 ± 0.06	3.3 ± 0.14	4.4 ± 0.22*

***R6/2-Chol or R6/2-untreated vs WT

Appendix Table S5 - chemico-physical characterization and drug content in the NPs in the preclinical trials

NPs	tyoe of experiments	Rhod	g7	Chol administered (mg/g)	Size (nm) (PDI)	Z-potential (mV)
g7-NPs	first pre-clinical trial (electrophysiology)	✓	✓	-	192 (0.06)	-11.7
g7-NPs-Chol		✓	✓	0,7	199 (0.06)	-12.5
g7-NPs	second pre-clinical trial (electrophysiology)	✓	✓	-	176 (0.09)	-12.2
g7-NPs-Chol		✓	✓	0,7	174 (0.08)	-9.1
g7-NPs	third preclinical trial (behaviour)	✓	✓	-	210 (0.11)	-5.7
g7-NPs-Chol		✓	✓	0,7	190 (0.08)	-9.2
g7-NPs	fourth preclinical trial (behaviour)	✓	✓	-	187 (0.09)	-6.4
g7-NPs-Chol		✓	✓	0,7	210 (0.12)	-7.3
g7-NPs	fifth preclinical trial (behaviour)	✓	✓	-	147 (0.09)	-16.4
g7-NPs-Chol		✓	✓	0,7	151 (0.10)	-17.3

Appendix Table S6 - summary all animals used in this study

	n° exp	date	Nps	n° WT mice	n° R6/2 mice	injection	n° injections	sacrifice after first injection	
characterization and distribution of g7-NPs	1	28/04/10	g7-NPs	2		iv	1	2 hrs	ip vs. iv injection<br>Nps kinetics in the mouse brain
			g7-NPs	4		iv	1	4 hrs	
			g7-NPs	3		iv	1	12 hrs	
			C-NPs	2		iv	1	2 hrs	
			C-NPs	2		iv	1	4 hrs	
			g7-NPs	2		ip	1	4 hrs	
			g7-NPs	2		ip	1	12 hrs	
	2	07/06/10	g7-NPs-NBD-Chol	3		ip	1	4 hrs	NPs kinetics in the mouse brain<br>NBD and g7-NPs co-localization<br>IHC different brain cells
			g7-NPs-NBD-Chol	3		ip	1	24 hrs	
			g7-NPs-NBD-Chol	3		ip	1	48 hrs	
			g7-NPs-NBD-Chol	3		ip	1	1 week	
			g7-NPs-NBD-Chol	2	2	ip	2	48 hrs	
			g7-NPs-NBD-Chol	2	2	ip	3	1 week	
			saline	2	2	ip	1	48 hrs	
			saline	2	2	ip	3	1 week	
	3	12/07/10	g7-NPs-NBD-Chol	1	3	ip	1	1 week	NPs kinetics in the mouse brain<br>first co-localization study
			g7-NPs-NBD-Chol		3	ip	1	2 weeks	
			g7-NPs-NBD-Chol		3	ip	1	3 weeks	
			saline	1	3	ip	1	3 weeks	
			g7-NPs-NBD-Chol	1	3	ip	2	2 weeks	
			g7-NPs-NBD-Chol	1	3	ip	3	3 weeks	
			saline	1	3	ip	3	3 weeks	
			g7-NPs-NBD-Chol	1		ip	1	5 months	
	4	08/07/12	g7-NPs	3	3	ip	1	4 hrs	IHC different brain cells<br>g7-NPs counts in liver, striatum and cortex
	5	01/07/12	empty-C-NPs	4	4	ip	2/week	5 weeks	effect of cholesterol supplementation only in periphery
			C-NPs-Chol	4	4	ip	2/week	5 weeks	
	6	01/08/12	g7-NPs-NBD-Chol		3	ip	1	24 hrs	IHC different brain cells; second co-localization study; g7-NPs counts in brain
			g7-NPs-NBD-Chol		3	ip	1	48 hrs	
			g7-NPs-NBD-Chol		3	ip	1	7 days	
			g7-NPs-NBD-Chol		3	ip	1	14 days	
	7	28/05/13	g7-NPs		2	ip	1	24 hrs	IHC different brain cells
			g7-NPs-NBD-Chol		2	ip	1	24 hrs, 1 week	
			saline	2	2	ip	1	24 hrs	
pre-clinical trials	8	01/10/10	g7-NPs	4	4	ip	3	5 weeks	first pilot study (UCLA), electrophysiological studies; rotarod test
			g7-NPs-Chol	10	10	ip	3	5 weeks	
	9	01/05/11	g7-NPs	5	5	ip	2/week	5 weeks	second pilot study (UCLA), for electrophysiological studies
			g7-NPs-Chol	5	5	ip	2/week	5 weeks	
			saline	5	5	ip	2/week	5 weeks	
	10	01/07/13	g7-NPs		3	ip	2/week	5 weeks	third pilot study (UNIMI), for behavioral tests (rotarod and NORT); neurophatology, biochemestry
			g7-NPs-Chol		6	ip	2/week	5 weeks	
			saline	8	7	ip	2/week	5 weeks	
	11	01/11/13	g7-NPs		4	ip	2/week	5 weeks	forth pilot study (UNIMI), for behavioral tests (rotarod, NORT, open field); neurophatology, biochemestry
			g7-NPs-Chol		8	ip	2/week	5 weeks	
			saline	8	8	ip	2/week	5 weeks	
	12	01/06/14	g7-NPs		6	ip	2/week	5 weeks	fifth pilot study (UNIMI), for behavioral tests (NORT, open field); neurophatology, biochemestry, qRTPCR (injections until 11 ws)
			g7-NPs-Chol		8	ip	2/week	5 weeks	
			saline	8	8	ip	2/week	5 weeks	

Appendix Table S7 - summary of statistics for the main figures

Figure	Statistical Analysis				
**Figure 2C**	unpaired t test				
	WT liver vs R6/2 liver				
	P value	0,0104			
	P value summary	*			
	Are means signif. different? (P < 0.05)	Yes			
	One- or two-tailed P value?	Two-tailed			
	t, df	t=2.859 df=18			
	WT cortex vs R6/2 cortex				
	P value	< 0.0001			
	P value summary	***			
	Are means signif. different? (P < 0.05)	Yes			
	One- or two-tailed P value?	Two-tailed			
	t, df	t=9.559 df=18			
	WT striatum vs R6/2 striatum				
	P value	< 0.0001			
	P value summary	***			
	Are means signif. different? (P < 0.05)	Yes			
	One- or two-tailed P value?	Two-tailed			
	t, df	t=8.309 df=18			

**Figure 3D**	One-way analysis of variance				
	P value	< 0.0001			
	P value summary	***			
	Are means signif. different? (P < 0.05)	Yes			
	Number of groups	4			
	F	12,34			
	R squared	0,5069			
	Bonferroni's Multiple Comparison Test	Mean Diff.	t	Significant? P < 0.05?	Summary
	brain 24h vs brain 48h	34,06	0,6415	No	ns
	brain 24h vs brain 7d	255,0	4,804	Yes	***
	brain 24h vs brain 14d	232,1	4,371	Yes	***
	brain 48h vs brain 7d	221,0	4,162	Yes	**
	brain 48h vs brain 14d	198,0	3,730	Yes	**
	brain 7d vs brain 14d	-22,97	0,4326	No	ns

**Figure 4B**	One-way analysis of variance				
	P value	< 0.0001			
	P value summary	***			
	Are means signif. different? (P < 0.05)	Yes			
	Number of groups	3			
	F	39,46			
	R squared	0,4315			
	Newman-Keuls Multiple Comparison Test	Mean Diff.	q	Significant? P < 0.05?	Summary
	WT (n=52) vs R6/2-untreated (n=27)	-6,793	12,39	Yes	***
	WT (n=52) vs R6/2-Chol (n=29)	-3,396	6,338	Yes	***
	R6/2-untreated (n=27) vs R6/2-Chol (n=29)	-3,397	5,514	Yes	*

**Figure 4E**	One-way analysis of variance				
	P value	< 0.0001			
	P value summary	***			
	Are means signif. different? (P < 0.05)	Yes			
	Number of groups	3			
	F	78,23			
	R squared	0,6197			
	Newman-Keuls Multiple Comparison Test	Mean Diff.	q	Significant? P < 0.05?	Summary
	WT (n=52) vs R6/2-untreated (n=27)	-1,990	15,37	Yes	***
	WT (n=52) vs R6/2-Chol (n=29)	-0,1656	1,047	No	ns
	R6/2-untreated (n=27) vs R6/2-Chol (n=29)	-1,824	13,09	Yes	***

**Figure 5B**	two-way ANOVA test				
	WT vs R6/2-untreated				
	time	Difference	t	P value	Summary
	5 ws	-22,27	1,107	P > 0.05	ns
	7 ws	-70,36	3,496	P<0.01	**
	9 ws	-95,94	4,768	P<0.001	***
	11 ws	-169,9	8,030	P<0.001	***
	WT vs R6/2-Chol				
	time	Difference	t	P value	Summary
	5 ws	-29,99	1,320	P > 0.05	ns
	7 ws	-79,95	3,518	P<0.01	**
	9 ws	-119,7	5,268	P<0.001	***
	11 ws	-179,0	7,878	P<0.001	***

**Figure 5C**	two-way ANOVA test				
	WT vs R6/2-untreated				
	treatment	Difference	t	P value	Summary
	5	-39,91	5,890	P<0.001	***
	10	-36,07	5,324	P<0.001	***
	15	-35,53	5,244	P<0.001	***
	20	-36,26	5,352	P<0.001	***
	25	-36,77	5,426	P<0.001	***
	30	-39,44	5,821	P<0.001	***
	35	-30,75	4,538	P<0.001	***
	40	-33,94	5,009	P<0.001	***
	45	-40,27	5,944	P<0.001	***
	50	-31,47	4,645	P<0.001	***
	55	-35,39	5,223	P<0.001	***
	60	-33,13	4,890	P<0.001	***

	WT vs R6/2-Chol				
	treatment	Difference	t	P value	Summary
	5	-39,08	5,241	P<0.001	***
	10	-27,82	3,732	P<0.01	**
	15	-29,22	3,919	P<0.01	**
	20	-30,65	4,111	P<0.001	***
	25	-33,11	4,441	P<0.001	***
	30	-36,49	4,895	P<0.001	***
	35	-29,34	3,935	P<0.01	**
	40	-31,36	4,207	P<0.001	***
	45	-38,03	5,101	P<0.001	***
	50	-27,25	3,655	P<0.01	**
	55	-31,71	4,254	P<0.001	***
	60	-28,32	3,798	P<0.01	**

**Figure 5D**	one-way ANOVA test				
	P value	< 0.0001			
	P value summary	***			
	Are means signif. different? (P < 0.05)	Yes			
	Number of groups	3			
	F	32,65			
	R squared	0,5763			
	Newman-Keuls Multiple Comparison Test	Mean Diff.	q	Significant? P < 0.05?	Summary
	R6/2-untreated vs WT	-3890	11,36	Yes	***
	R6/2-untreated vs R6/2-Chol	-1133	3,379	Yes	*
	R6/2-Chol vs WT	-2757	7,408	Yes	***
**Figure 5E**					
NORT - 8 weeks	One-way analysis of variance				
	P value	< 0.0001			
	P value summary	****			
	Are means signif. different? (P < 0.05)	Yes			
	Number of groups	3			
	F	17,42			
	R square	0,3087			
	Newman-Keuls Multiple Comparison Test	Mean Diff.	q	Significant? P < 0.05?	Summary
	R6/2-untreated vs WT	-38,54	8,271	Yes	***
	R6/2-untreated vs R6/2-Chol	-20,46	4,213	Yes	**
	R6/2-Chol vs WT	-18,09	3,423	Yes	*
NORT - 10 weeks	One-way analysis of variance				
	P value	< 0.0001			
	P value summary	****			
	Are means signif. different? (P < 0.05)	Yes			
	Number of groups	3			
	F	22,32			
	R square	0,3669			
	Newman-Keuls Multiple Comparison Test	Mean Diff.	q	Significant? P < 0.05?	Summary
	R6/2-untreated vs WT	-48,29	8,904	Yes	***
	R6/2-untreated vs R6/2-Chol	-37,02	6,377	Yes	***
	R6/2-Chol vs WT	-11,27	1,813	No	ns
NORT - 12 weeks	One-way analysis of variance				
	P value	< 0.0001			
	P value summary	****			
	Are means signif. different? (P < 0.05)	Yes			
	Number of groups	3			
	F	18,27			
	R square	0,3430			
	Newman-Keuls Multiple Comparison Test	Mean Diff.	q	Significant? P < 0.05?	Summary
	R6/2-untreated vs WT	-48,22	8,299	Yes	***
	R6/2-untreated vs R6/2-Chol	-33,04	5,311	Yes	***
	R6/2-Chol vs WT	-15,18	2,330	No	ns

**Figure 6A**					
PSD95	One-way analysis of variance				
	P value	< 0.0001			
	P value summary	***			
	Are means signif. different? (P < 0.05)	Yes			
	Number of groups	3			
	F	36,39			
	R squared	0,5740			
	Newman-Keuls Multiple Comparison Test	Mean Diff.	q	Significant? P < 0.05?	Summary
	R62-untreated vsWT	-0,2700	11,60	Yes	***
	R62-untreated vs R6/2-Chol	-0,1973	7,916	Yes	***
	R6/2-Chol vs WT	-0,07268	2,802	No	ns
Gephryin	One-way analysis of variance				
	P value	0,0739			
	P value summary	ns			
	Are means signif. different? (P < 0.05)	No			
	Number of groups	3			
	F	2,873			
	R squared	0,1755			
	Newman-Keuls Multiple Comparison Test	Mean Diff.	q	Significant? P < 0.05?	Summary
	R62-untreated vsWT	-0,1670	3,161	No	ns
	R62-untreated vs R6/2-Chol	-0,1409	---	No	ns
	R6/2-Chol vs WT	-0,02604	---	No	ns

GluN1	One-way analysis of variance				
	P value	< 0.0001			
	P value summary	***			
	Are means signif. different? (P < 0.05)	Yes			
	Number of groups	3			
	F	16,54			
	R squared	0,5416			
	Newman-Keuls Multiple Comparison Test	Mean Diff.	q	Significant? P < 0.05?	Summary
	R62-untreated vsWT	-0,6375	8,111	Yes	***
	R62-untreated vs R6/2-Chol	-0,2169	2,760	No	ns
	R6/2-Chol vs WT	-0,4206	4,923	Yes	**
GluN2B	One-way analysis of variance				
	P value	0,0028			
	P value summary	**			
	Are means signif. different? (P < 0.05)	Yes			
	Number of groups	3			
	F	7,188			
	R squared	0,3240			
	Newman-Keuls Multiple Comparison Test	Mean Diff.	q	Significant? P < 0.05?	Summary
	R62-untreated vs R6/2-Chol	-0,3574	4,921	Yes	**
	R62-untreated vsWT	-0,2780	4,051	Yes	**
	R6/2-Chol vs WT	-0,07939	1,055	No	ns
beta-3-tub	One-way analysis of variance				
	P value	0,1550			
	P value summary	ns			
	Are means signif. different? (P < 0.05)	No			
	Number of groups	3			
	F	1,956			
	R squared	0,09117			
	Newman-Keuls Multiple Comparison Test	Mean Diff.	q	Significant? P < 0.05?	Summary
	R62-untreated vsWT	-0,1177	2,796	No	ns
	R62-untreated vs R6/2-Chol	-0,05825	---	No	ns
	R6/2-Chol vs WT	-0,05946	---	No	ns

**Figure 6C**	R6/2-Chol vs R6/2-untreated				
	bdnf cortex				
	Unpaired t test				
	P value	0,0001			
	P value summary	***			
	Are means signif. different? (P < 0.05)	Yes			
	One- or two-tailed P value?	Two-tailed			
	t, df	t=4.475 df=25			
	bdnf hippocampus				
	Unpaired t test				
	P value	0,1607			
	P value summary	ns			
	Are means signif. different? (P < 0.05)	No			
	One- or two-tailed P value?	Two-tailed			
	t, df	t=1.441 df=28			

**Figure 6D**	snap25 cortex				
	Unpaired t test				
	P value	0,0092			
	P value summary	**			
	Are means signif. different? (P < 0.05)	Yes			
	One- or two-tailed P value?	Two-tailed			
	t, df	t=2.920 df=18			
	snap25 hippocampus				
	Unpaired t test				
	P value	0,0729			
	P value summary	ns			
	Are means signif. different? (P < 0.05)	No			
	One- or two-tailed P value?	Two-tailed			
	t, df	t=1.905 df=18			

**Figure 6E**	complexin cortex				
	Unpaired t test				
	P value	0,1774			
	P value summary	ns			
	Are means signif. different? (P < 0.05)	No			
	One- or two-tailed P value?	Two-tailed			
	t, df	t=1.404 df=18			
	complexin hippocampus				
	Unpaired t test				
	P value	0,0169			
	P value summary	*			
	Are means signif. different? (P < 0.05)	Yes			
	One- or two-tailed P value?	Two-tailed			
	t, df	t=2.666 df=16			
	complexin striatum				
	Unpaired t test				
	P value	0,0151			
	P value summary	*			
	Are means signif. different? (P < 0.05)	Yes			
	One- or two-tailed P value?	Two-tailed			
	t, df	t=2.687 df=18			

**Figure 6G**	One-way analysis of variance				
	P value	0,0180			
	P value summary	*			
	Are means signif. different? (P < 0.05)	Yes			
	Number of groups	4			
	F	4,071			
	R squared	0,3372			
	Newman-Keuls Multiple Comparison Test	Mean Diff.	q	Significant? P < 0.05?	Summary
	R6/2-emp vs R6/2	-541900000	4,141	Yes	*
	R6/2-emp vs R6/2-Chol	-92100000	0,7250	No	ns
	R6/2-emp vs WT	-18000000	---	No	ns
	WT vsR6/2-Chol	-74100000	---	No	ns
	R6/2-Chol vs R6/2	-449800000	3,695	Yes	*

**Figure 7A**	One-way analysis of variance				
	P value	< 0.0001			
	P value summary	***			
	Are means signif. different? (P < 0.05)	Yes			
	Number of groups	3			
	F	79,31			
	R squared	0,9243			
	Newman-Keuls Multiple Comparison Test	Mean Diff.	q	Significant? P < 0.05?	Summary
	r62 chol vs wt saline	-21,12	13,53	Yes	***
	r62 chol vs r62 saline	-1,197	0,7860	No	ns
	r62 saline vs wt saline	-19,92	16,22	Yes	***
**Figure 7B**	One-way analysis of variance				
	P value	0,0016			
	P value summary	**			
	Are means signif. different? (P < 0.05)	Yes			
	Number of groups	3			
	F	11,02			
	R squared	0,6290			
	Newman-Keuls Multiple Comparison Test	Mean Diff.	q	Significant? P < 0.05?	Summary
	R6/2-Chol vs WT	-4,237	5,916	Yes	**
	R6/2-Chol vs R6/2	-0,3880	0,5187	No	ns
	R6/2 vs WT	-3,849	5,374	Yes	**

**Figure 7E**	One-way analysis of variance				
	P value	< 0.0001			
	P value summary	***			
	Are means signif. different? (P < 0.05)	Yes			
	Number of groups	4			
	F	24,89			
	R squared	0,5586			
	Newman-Keuls Multiple Comparison Test	Mean Diff.	q	Significant? P < 0.05?	Summary
TNF-alpha	WT vs R6/2-emp	-3,087	9,294	Yes	***
	WT vs R6/2-Chol	-2,386	8,344	Yes	***
	WT vs R6/2	-2,339	8,820	Yes	***
IL6	WT vs R6/2-emp	-0,7488	2,064	No	ns
	WT vs R6/2-Chol	-0,04721	---	No	ns
	WT vs R6/2	-0,7016	---	No	ns
**Figure 7F**	One-way analysis of variance				
	P value	< 0.0001			
	P value summary	***			
	Are means signif. different? (P < 0.05)	Yes			
	Number of groups	4			
	F	11,98			
	R squared	0,3786			
	Newman-Keuls Multiple Comparison Test	Mean Diff.	q	Significant? P < 0.05?	Summary
TNF-alpha	WT vs R6/2	-1,049	7,972	Yes	***
	WT vs R62-emp	-0,8024	4,866	Yes	**
	WT vs R6/2-Chol	-0,5491	3,869	Yes	**
IL6	R6/2-Chol vs R6/2	-0,5000	3,139	No	ns
	R6/2-Chol vs R62-emp	-0,2533	---	No	ns
	R62-emp vs R6/2	-0,2467	---	No	ns

Appendix Methods

Details about formulation and characterization of NPs employed in this study are herein described.

Purification and collection of the NPs

To purify the loaded NPs from the unloaded Chol, NBD-Chol and PVA residuals, all the samples were centrifuged at 17,000 rpm for 10 min (Sorvall RC28S, Dupont, Brussels, Belgium), washed several times with water and re-suspended in water. The purified NP suspensions were freeze-dried (-60°C, $1\cdot10^{-3}$ mm/Hg, for 48 h; LyoLab 3000, Heto-Holten, Allerod, Denmark) using trehalose as cryoprotectant (1:0.5 w:w polymer/trehalose ratio). After the liophylisation process, all the samples were stored at 4°C in N_2 atmosphere. The yield of NPs (Yield %) was calculated as the percentage of NPs recovered after the freeze-drying procedure compared to the weight of PLGA and Chol used for the preparation as follows: Yield (%) = (NPs recovered after the freeze-dried process – anidre trehalose - PVA residual) weight x100/ (PLGA+Chol) weight. Before their use, freeze-dried NPs were weighed, re-suspended in water at the concentration of 10 mg/mL by bath sonication for 3 min at r.t.

Determination of the amount of PVA residual

As the residual PVA associated with the NPs could affect the physical properties and the uptake by cells, the residual PVA was determined by a colorimetric method based on the formation of the colored complex between two adjacent hydroxyl groups of PVA and an iodine molecule. Briefly, freeze-dried NPs (5 mg) were solubilized in dichloromethane (1 mL). Then, water (2 mL) was added and the organic solvent was evaporated at r.t. under stirring (2 h). The suspension was filtered (cellulose nitrate filter, porosity 0.45 µm, Sartorius, Florence, Italy) to remove the polymeric residue and the aqueous solution (1 mL) was treated with 0.5 M NaOH (2 mL) for 15 min at 60°C. The solution was neutralized with 1 N HCl (900 µL) and the volume adjusted to 5 mL with water. Then, a solution of I_2/KI (0.5 mL) (0.05 M/0.15 M) and water (1.5 mL) was added to a solution of boric acid (0.65 M; 3 mL). PVA concentration was determined measuring the absorbance at 690 nm after 15 min of incubation at r.t. in comparison with a standard plot of PVA prepared under the same experimental conditions.

Chemico-physical characterization

The surface morphology of both unloaded and loaded NPs was evaluated by means of the atomic force microscope (AFM, Park Instruments, Sunnyvale, CA, USA) analysis at r.t. (about 25°C) operating in air and in non-contact mode using triangular silicon tips. The resonant frequencies of the cantilever were found to be about 160 kHz. Before the analysis, a drop (20 µL) of a water-diluted suspension of the NPs (about 0.01 mg/mL) was applied on a small mica disk (1 cm × 1 cm); after 2 min, the excess of water was removed using paper filter. The topographical images obtained, also called "height" images, were flattened using second-order fitting to remove sample tilt.

Architecture and internal structure of NPs was studied by using the transmission electron microscope (TEM) operating at an acceleration voltage of 200 KV (model JEM 2010; JEOL, Oxford Instruments, Abingdon, England). Briefly, a drop of a water-diluted suspension of the samples (about 0.03 mg/mL) was placed on a 200-mesh copper grid (TABB Laboratories Equipment, Berks, UK), allowed to adsorb and the suspension surplus was removed by filter paper.

Mean particle size (Z-Average) and polydispersivity index (PDI) of the NPs (in distilled water) were determined at 25°C by PCS using a Zetasizer Nano ZS (Malvern, UK; Laser 4 mW He–Ne, 633 nm, Laser attenuator Automatic, transmission 100–0.0003%, Detector Avalanche photodiode, Q.E. > 50% at 633 nm, T = 25 °C). The results were normalized with respect to a polystyrene standard suspension. The zeta potential (ζ-pot) was measured by using the same

equipment with a combination of laser Doppler velocimetry and phase analysis light scattering (PALS). All the data are expressed as means of at least three determinations carried out for each preparation lot (three lots for each sample).

Drug entrapment efficiency (EE%) and loading capacity (LC%)

To quantify the amount of Chol loaded into NPs, an exact amount of loaded NPs (5 mg) was dissolved in chloroform (0.5 mL). Then, isopropyl alcohol (1mL) (in which Chol, but not PLGA, is soluble) was added. The mixture was then vortexed (15 Hz for 1 min; ZX3, VelpScientifica, Usmate, Italy) to promote the precipitation of the polymer and then filtered (polytetrafluoroethylene filter, porosity 0.20 μm, Sartorius). The amount of Chol loaded in the NPs was quantified by RP-HPLC. The HPLC apparatus (JASCO Europe, Cremella, Italy) comprised a Model PU980 pump provided with an injection valve with a 50 μL sample loop (Jasco, Model 7725i) and the UV detector (Jasco UV975). Chromatography separation was carried out on a Syncronics C18 (250x4.6 mm; porosity 5 μm; Thermo Fisher Scientific, Waltham, MA, USA) at r.t., flow rate of 1.2 mL/min, by operating in an isocratic mode using 50:50 v/v acetonitrile:ethanol as mobile phase. Before the use, mobile phase eluents were filtered through a 0.45 μm hydrophilic polypropylene membrane filters (Sartorius). The eluent absorbance was monitored at 220 nm using the UV detector (Jasco UV975). Chromatographic peak area of the standard solution were collected and used for the generation of calibration curve. Linearity was assumed in the range of 18-300 μg/mL (r^2=0.995).

The entrapment efficiency (EE) and the loading capacity (LC), expressed as percentage, were calculated using the following formula: EE%= D/Td×100; LC%= D/W×100 where D is the amount of Chol loaded in the NPs, Td is the amount of drug used for the preparation and W is the weight of the NPs (polymer + drug). All the data are expressed as the mean of at least three determinations.

Release of Chol and NBD-Chol from loaded NPs under simulated conditions

Figure 1D shows the % of Chol and NBD-Chol released in water. The initial "burst effect" can be attributed to the fraction of Chol adsorbed or in close contact with the surface of the NPs. This surface Chol fraction dissolved into the surrounding liquid, leading to the fast initial release profile. Moreover, during the second phase, Chol embedded in the NPs could significantly delay the *in vitro* release. The slow linear release kinetic of Chol from NPs-Chol between day 5 and day 10 could be ascribed to NPs degradation. While hydrophilic drugs with high solubility could increase the rate of water diffusion into the biodegradable matrix, accelerating the release and the (bulk) erosion, hydrophobic molecules with low water solubility (as Chol) may hinder water diffusion into the matrix, slowing the release rate as the consequence of the NPs surface erosion with a consequent release.

An exact amount of lyophilized NPs-Chol1 or NPs-NBD-Chol1 (0.5 mg) were suspended in water under perfect sink conditions (6 mL) into a well closed glass vial that was incubated into a water bath at 37±0.2°C under magnetic stirring (250 rpm). At fixed time intervals, the loaded NPs were centrifuged (40,000 rpm for 30 min at 10°C) and the surnatant (1 mL) was collected to be lyophilized (-60°C, $1 \cdot 10^{-3}$ mm/Hg, for 48 h; LyoLab 3000, Heto-Holten). To evaluate the amount of Chol released from NPs-Chol1, the freeze dried residual was dissolved in water and derivatized with 50 μL of N,O-bis(trimethylsilyl) trifluoroacetamide/trimethylchlorosilane (BSTFA/TMCS) (Sigma–Aldrich).The mixture was heated at 60°C for 30 min. Then, the sample was equilibrated at 25°C and diluted with n-exane. The quantification of the derivatized-Chol was performed by gas chromatography (GC) with selected ion monitoring mass spectrometric (SIM-MSD) using Agilent (Santa Clara, CA, USA) 7890A gas chromatography interfaced with an 5975C Series GC/MSD with Triple-Axis HED-EM Detector and equipped with an Agilent 190915-433 column (30m*250μm*0.25μm). The chromatographic conditions were as follow: initial oven temperature 240°C which was

increased by 15°C/min to 315°C. The temperature of the injector was set at 280°C. Helium was used as the carrier gas in a splitless mode injection (3 μL). In selected ion monitoring (SIM) mode three characteristic ions were analyzed (329, 353 and 368 m/z) and the most representative 329 m/z was used for the calibration.

Calibration was achieved starting from stock solution of Chol (1mg/mL) properly diluted with chloroform, evaporated to dryness and analyzed in the manner previously described. Linearity was assured in the range 0.1-2.7 ppm (r^2=0.998).

To evaluate the amount of NBD-Chol released from NPs-NBD-Chol1, the lyophilized residual was solubilized in 1:5 v/v chloroform:isopropyl alcohol mixture and injected in a chromatographic system. The HPLC apparatus (Jasco, Model PU 2089 pump, injection valve model 7725i with a 20 µl sample loop) was correlated with a fluorimetric detector (Jasco FP 2020). Chromatographic separation was carried out on a Syncronis C18 column (250*4.6 µm; 5µm; ThermoScientific) at 30°C and at a flow-rate of 1 mL/min. Elution was performed in an isocratic mode using as mobile phase a 90:10 v/v acetonitrile:isopropyl alcohol mixture. Mobile phase eluents were filtered through a 0.45 µm of hydrophilic polypropylene membrane filters (Sartorius) before the use. The fluorescence of the eluent was monitored by excitation at 484 nm detecting the emission at 539 nm. A calibration curve was done by analyzing standard solution assuring linearity in the range 0.4-16 µg/ml (r^2=0.995).

In vitro release studies and NBD-Chol quantification

For quantitative studies, NS cells were plated in proliferation medium in 6-mwell plates at 300,000 cells/well. Cells were treated for different time points (24-48-72 h) with 165 µL NPs-NBD-Chol1 suspension (4 mg/mL, 440 µg NBD-Chol/100mg NPs) to receive 3 µg NBD-Chol. After the treatment, the cells were washed with PBS, collected in tubes and centrifuged for 5 min at 1000xg. The centrifuged cell pellets were re-suspended in 400 µl of Lysis Buffer [10 mM TRIS HCl pH 7.5, 150 mM NaCl, 5 mM EDTA, supplemented with PMSF (phenylmethylsulfonyl fluoride) 1:200 and Protease inhibitor 1:100] to obtain a homogenate containing total NBD-Chol (released and still encapsulated into NPs). A fraction of the homogenate (200 µL) was centrifuged for 15 min at 19000xg to separate NBD-Chol still encapsulated into NPs (pellet) from NBD-Chol released after NPs degradation (surnatant). NBD-Chol was quantified in duplicate in both fractions (pellet and surnatant) and in total homogenate by spectrophotometry at 488 nm. NBD-Chol release was calculated by subtracting the NBD fluorescence detected in the pellet (containing the intact NPs) to NBD fluorescence detected in the homogenate (total NBD-Chol present into cells).
