# Supplementary material for: Cholesterol‐loaded nanoparticles ameliorate synaptic and cognitive function in Huntington's disease mice
Source: EMBO Mol Med. 2015 Nov 20;7(12):1547–64. doi: 10.15252/emmm.201505413 (PMC4693506; doi:10.15252/emmm.201505413)
Supplement: Supplementary file 3 — Source Data for Figure 6 [file EMMM-7-1547-s002.pdf]

Source Data_Figure 6– original, uncropped and unprocessed scans of the gels in Fig. 6A

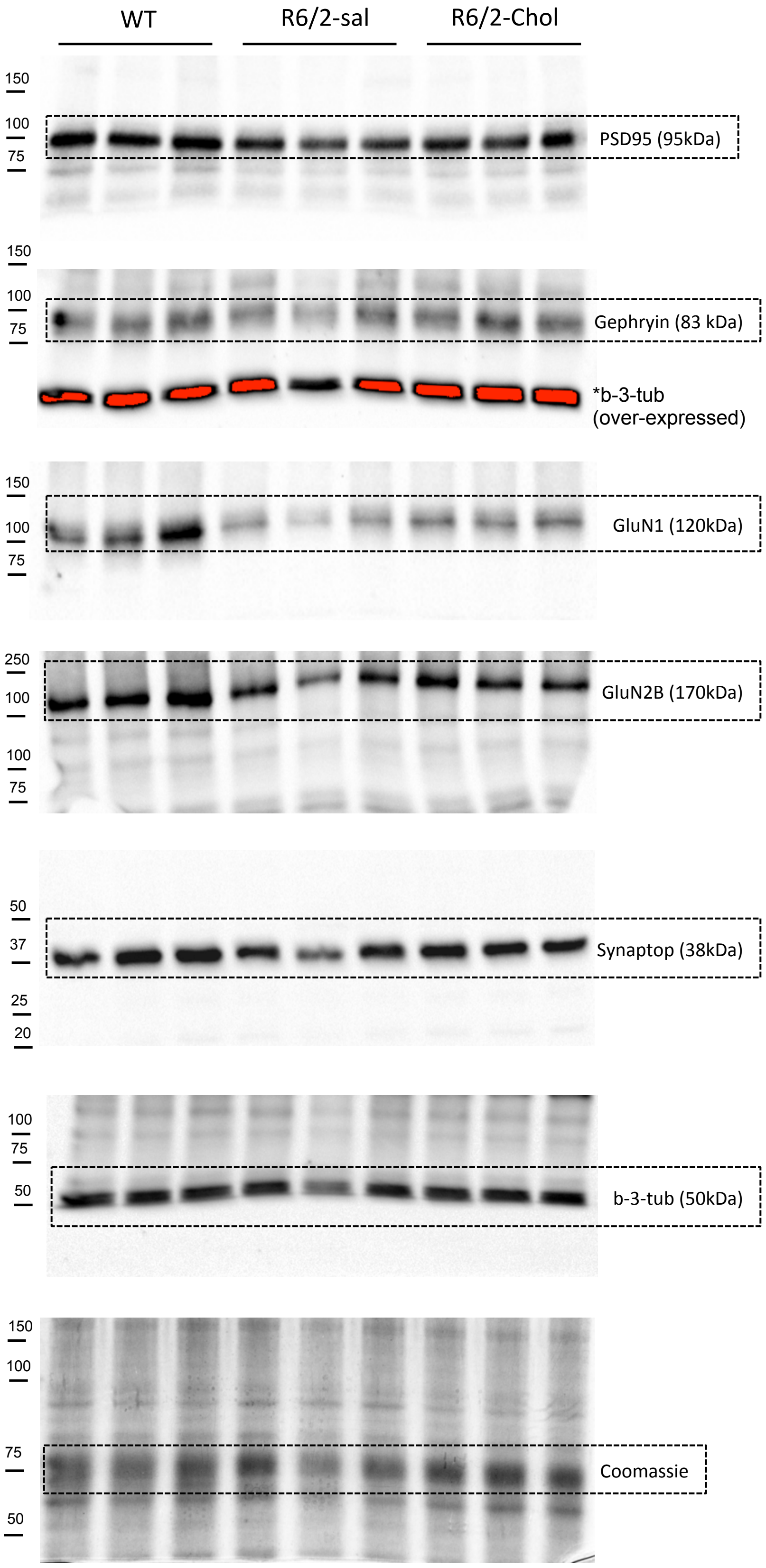

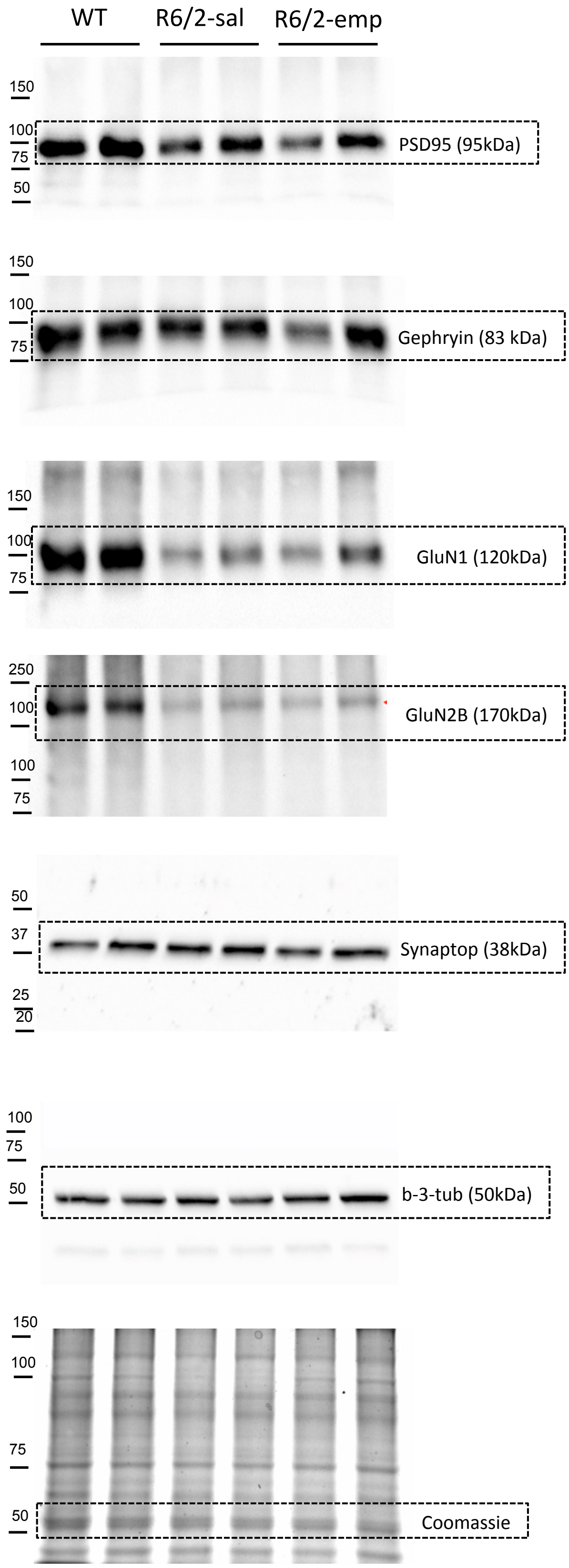

Source Data_Figure 6A. Full-length pictures of the plots presented in Figure 6A. Dashed boxes indicate the bands shown in Figure 6A. In some cases, when it was possible, blots were cut first and then probed with different antibodies.
